# Single-Prolonged Stress Impairs Prefrontal Cortex Control of Amygdala and Striatum in Rats

**DOI:** 10.3389/fnbeh.2019.00018

**Published:** 2019-04-30

**Authors:** Veronica M. Piggott, Kelly E. Bosse, Michael J. Lisieski, John A. Strader, Jeffrey A. Stanley, Alana C. Conti, Farhad Ghoddoussi, Shane A. Perrine

**Affiliations:** ^1^Research & Development Service, John D. Dingell VA Medical Center, Detroit, MI, United States; ^2^Department of Neurosurgery, Wayne State University School of Medicine, Detroit, MI, United States; ^3^Department of Psychiatry and Behavioral Neurosciences, Wayne State University School of Medicine, Detroit, MI, United States; ^4^Department of Anesthesiology, Wayne State University School of Medicine, Detroit, MI, United States

**Keywords:** post-traumatic stress disorder, single-prolonged stress, ^1^H-MRS, MEMRI, striatum, amygdala, prelimbic cortex, infralimbic cortex

## Abstract

Medial prefrontal cortex (mPFC), amygdala, and striatum neurocircuitry has been shown to play an important role in post-traumatic stress disorder (PTSD) pathology in humans. Clinical studies show hypoactivity in the mPFC and hyperactivity in the amygdala and striatum of PTSD patients, which has been associated with decreased mPFC glutamate levels. The ability to refine neurobiological characteristics of PTSD in an animal model is critical in furthering our mechanistic understanding of the disease. To this end, we exposed male rats to single-prolonged stress (SPS), a validated model of PTSD, and hypothesized that traumatic stress would differentially activate mPFC subregions [prelimbic (PL) and infralimbic (IL) cortices] and increase striatal and amygdalar activity, which would be associated with decreased mPFC glutamate levels. *in vivo*, neural activity in the subregions of the mPFC, amygdala, and striatum was measured using manganese-enhanced magnetic resonance imaging (MEMRI), and glutamate and N-acetylaspartate (NAA) levels in the mPFC and the dorsal striatum (dSTR) were measured using proton magnetic resonance spectroscopy (^1^H-MRS) longitudinally, in rats exposed to SPS or control conditions. As hypothesized, SPS decreased MEMRI-based neural activity in the IL, but not PL, cortex concomitantly increasing activity within the basolateral amygdala (BLA) and dorsomedial striatum (dmSTR). ^1^H-MRS studies in a separate cohort revealed SPS decreased glutamate levels in the mPFC and increased NAA levels in the dSTR. These results confirm previous findings that suggest SPS causes mPFC hypoactivation as well as identifies concurrent hyperactivation in dmSTR and BLA, effects which parallel the clinical neuropathology of PTSD.

## Introduction

Dysfunction of “top-down” prefrontal cortex (PFC) control in post-traumatic stress disorder (PTSD) likely contributes to amygdala hyperactivity, which is thought to mediate disease characteristics, such as the inability to inhibit fear-related behaviors related to traumatic events (Shin and Liberzon, 2010). Specifically, the dorsal anterior cingulate cortex (ACC) and dorsolateral PFC [analogous to the rodent prelimbic (PL) cortex] are implicated in regulating acquisition and expression of conditioned fear behaviors by activating the basolateral amygdala (BLA). In contrast, the ventromedial PFC [analogous to the rodent infralimbic (IL) cortex] is known to inhibit fear response by regulating intercalated cells of the amygdala to attenuate central nucleus of the amygdala (CeA) output (Sierra-Mercado et al., 2011; Do-Monte et al., 2015). Functional neuroimaging studies in humans with PTSD consistently show an increase activity in dorsal ACC and a decrease activity in ventromedial PFC, which is concomitant with an increase in amygdala activity (Hayes et al., 2012). Fear conditioning studies in animals have helped define this circuitry (Milad et al., 2006); however, recapitulation of these neuronal (or neurochemical) changes in animal models of PTSD has not been reported. Using proton magnetic resonance spectroscopy (^1^H-MRS), we and others have shown that glutamate (Glu) levels are decreased in the medial PFC (mPFC) of rodents exposed to the single-prolonged stress (SPS) model of PTSD (Knox et al., 2010; Perrine et al., 2016; Lim et al., 2017), consistent with human ^1^H-MRS studies in the ACC (Yang et al., 2015). Considering these parallels (Pitman et al., 2012), the present study aimed to confirm and extend our previous ^1^H-MRS findings by using manganese-enhanced magnetic resonance imaging (MEMRI) to quantify calcium-dependent neural activity in mPFC and amygdala, longitudinally, before and after SPS.

The PFC also influences striatal (STR) neural activity to regulate goal-directed, motivated, and habit behaviors (Peters et al., 2009; Everitt and Robbins, 2016). Importantly, a lack of inhibitory control in individuals with PTSD is associated with increased activation in the STR during a response inhibition test (Falconer et al., 2008) and with reduced striatal activation reward processing and responsivity (Sailer et al., 2008; Elman et al., 2009). PFC-STR projections have been identified in animals and, similar to the amygdala, the STR receives distinct glutamatergic mPFC input. The PL projects to the ventral STR, including the nucleus accumbens core and dorsomedial STR (dmSTR) to promote goal-directed behaviors, whereas the IL projects to the nucleus accumbens shell to inhibit goal-directed behaviors and to the dorsolateral STR (dlSTR) where its input stimulates the transition of goal-directed to habit-based behaviors (Peters et al., 2009; Everitt and Robbins, 2016). This neurocircuitry has been well-defined in the context of drug addiction and feeding behaviors, but the study of its role in traumatic stress responses is limited. Our findings using the SPS model show neurochemical changes in the STR, implicating it in anhedonia and cross-sensitization with drugs of abuse that relate to trauma exposure (Eagle et al., 2013, 2015; Enman et al., 2015; Matchynski-Franks et al., 2016). Therefore, a second goal of this study is to use longitudinal ^1^H-MRS and MEMRI to assess glutamatergic tone and neural activity, respectively, in subregions of the STR before and after SPS.

Here, we used SPS to evaluate the effects of traumatic stress exposure on neurochemistry and neural activity within the mPFC, amygdala, and STR. SPS has been shown to be a valid rodent model of PTSD displaying many of the expected characteristics observed in individuals with PTSD, including hyperarousal (Khan and Liberzon, 2004; Ganon-Elazar and Akirav, 2012), avoidance of aversive cues (Brand et al., 2008), emotional and cognitive deficits (Wang et al., 2008, 2010; Li et al., 2010; Eagle et al., 2013), and increased alcohol drinking (Blanco et al., 2013; Matchynski-Franks et al., 2016). Particularly important in relation to the present study, animals exposed to SPS show a deficit in the retention of extinction learning in conditioned fear paradigms (Milad et al., 2006; Knox et al., 2012a), which may indicate impairment in the IL-amygdala pathway that regulates extinction learning. We hypothesized that, at a post-trauma interval during which behavioral deficits are typically apparent in the SPS model, SPS-exposed animals would show: (1) decreased glutamate levels in the mPFC; (2) augmented neural activity in the PL and attenuated activity in the IL; (3) increased neural activity in the BLA; and (4) increased neural activity in the medial STR. To test these hypotheses, we collected neuroimaging data using ^1^H-MRS and MEMRI before and after SPS or control procedures, which allowed us to quantify Glu levels (Knox et al., 2012a) and neural activity (Perrine et al., 2015; Bosse et al., 2018), respectively, in a longitudinal *in vivo* design.

## Materials and Methods

### Experimental Design

Two experiments were conducted using separate cohorts of rats to determine the region-specific effects of SPS on brain neurochemistry and neural activity using ^1^H-MRS or MEMRI *in vivo*. In the first experiment, single-voxel ^1^H-MRS data at 7T were acquired in two regions of interest (ROIs): the mPFC and dorsal STR (dSTR) to measure Glu as well as N-acetylaspartate (NAA), a marker of neuronal integrity. Spectra were collected before and after SPS or control treatment; prescan measurements occurred between 2 and 96 h before exposure and postscan measurements occurred on day 8 or 9 after exposure to SPS. In the second experiment, MEMRI was conducted 7 days prior to SPS to obtain baseline measurements and again 20 days following SPS. MEMRI data were used to assess neural activity in the mPFC (PL and IL), amygdala (BLA and CeA), and dSTR (dmSTR and dlSTR) before and after SPS or control treatment. Also in the second experiment, an object-location memory task was performed 7 days after SPS or control conditions but data are not included as the behavioral task is not regulated by the neurocircuitry being studied herein, furthermore the results showed that no significant differences between groups.

### Animals

Thirty-six male Sprague-Dawley rats (Charles River Laboratories, Wilmington, MA, USA) weighing >240 g at the beginning of the study were pair-housed in standard microisolator cages upon arrival. Animals were acclimated to the climate-controlled vivarium under a 12-h light/dark cycle (lights on at 7 AM) for 5 days and handled once daily for 3 days prior to initiation of procedures. Stress exposure and neuroimaging experiments were conducted during the light phase of the light/dark cycle. Rats were allowed *ad libitum* access to standard rat chow and water except during experiments. Each day, animals were transported from the vivarium to the laboratory and habituated for at least 1 h before beginning experiments. All procedures were approved by the Wayne State University Institutional Animal Care and Use Committee and abided by guidelines in the Guide for the Care and Use of Laboratory Animals.

### Single-Prolonged Stress (SPS)

Rats were exposed to SPS as previously described (Knox et al., 2010; Eagle et al., 2015); for a review of the SPS model see (Lisieski et al., 2018). Rats were first restrained for 2-h in cylindrical clear plastic restraints. Immediately following this restraint, they were put into a large tub (48 cm top diameter) that was filled to a depth of 30 cm of room temperature water for a 20-min forced group swim (6–10 rats at a time). Following the swim, rats were towel-dried and given a 15-min rest period in a clean cage with fresh bedding. Finally, rats were exposed to diethyl ether vapor as a group (6–10 rats at a time) until loss of consciousness, as confirmed by absence of righting reflex and lack of response to toe and tail pinch (<5 min). Control animals were held in a separate room for an equivalent period of time, during which they were weighed and handled for ~2 min. Following SPS or control exposure, rats were returned to the vivarium and left undisturbed (except for routine animal care) for 7 days. This 7-day “incubation period” has been shown to be necessary for the development of PTSD-like effects on behavior (Knox et al., 2012b) and neuroendocrine markers (Liberzon et al., 1997, 1999) that model characteristics observed in PTSD.

### Proton-Magnetic Resonance Spectroscopy (^1^H-MRS)

**Figure 1:**
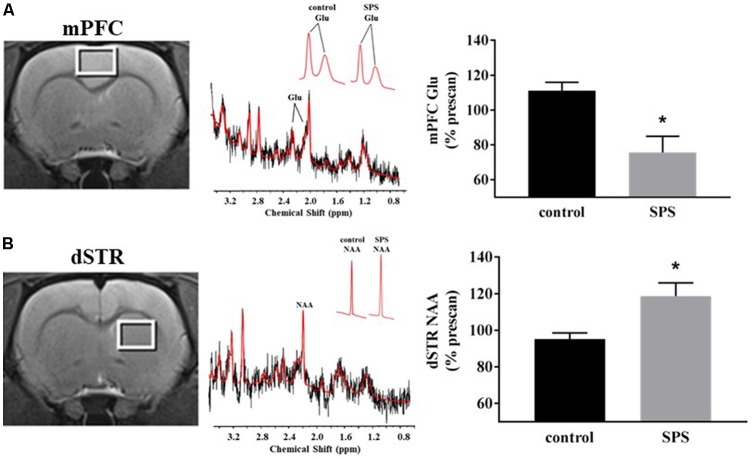
Effects of single prolonged stress (SPS) on glutamate (Glu) and N-acetylaspartate (NAA) levels in the medial prefrontal cortex (mPFC) and dorsal striatum (dSTR). **(A)** Left, voxel placement in the mPFC (white box, voxel size 3 mm × 2 mm × 3 mm). Middle, representative spectrum from the mPFC [Inset: Glu spectrum (peaks at 2.10 ppm and 2.35 ppm) from (Left) a control rat and (Right) an SPS-exposed rat]. Right, SPS decreased Glu levels relative to prescan levels in the mPFC (control *n* = 5, SPS *n* = 4) compared to controls. **(B)** Left, voxel placement in the dSTR (white box, voxel size 3 mm × 2 mm × 3 mm). Middle, representative spectrum from the dSTR [Inset: NAA spectrum from (Left) a control rat and (Right) an SPS-exposed rat]. Right, SPS increased NAA levels relative to prescan levels in the dSTR (control *n* = 4, SPS *n* = 5) compared to controls. Data are plotted as % prescan and expressed as mean ± SEM. *p < 0.05 compared to controls.

^1^H-MRS spectra were acquired on a 7T Bruker ClinScan system with a Siemens console using a transmit-only whole-body coil and receive-only surface coil. Prior to each scan, animals were anesthetized with isoflurane (1%–5%, 0.4 L/min O_2_) and maintained on the same percent of isoflurane through each scan. Animals were placed in a prone position and secured using blunted ear-bars and a tooth-bar before being placed into the magnet’s horizontal bore, and rats were warmed throughout scanning using a heated water-circulation system. Voxels (3 mm × 2 mm × 3 mm) were placed in mPFC and dSTR ROI. For mPFC, the voxels were placed at the center approximately +2.0 mm from Bregma, on the midline, with the bottom edge just superior to corpus callosum (Figure 1A, left). For dSTR, the voxels were placed at the center, approximately at Bregma, 3 mm from the midline, with the top corners just inferior to corpus callosum (Figure 1B, left). These placements were based on the rat brain atlas of Paxinos and Watson (Paxinos and Watson, 2007). Prior to each measurement, magnetic field homogeneity (a.k.a. shimming) over the voxel was adjusted to yield a water spectrum line width of 30–50 Hz using FASTESTMAP (Fast, Automatic Shim Technique using Echo-planar Signal readouT for Mapping Along Projections; Gruetter and Tkác, 2000). A PRESS sequence (repetition time = 4,000 ms, echo time = 3 ms, spectral width = 4 kHz; 2,048 data points; and at least 256 averages) was used to obtain spectra. Additionally, unsuppressed water spectra were acquired per animal for absolute metabolite quantification. The spectral data were analyzed using LCModel and with a basis set derived from simulated data. Only Cramér–Rao measurements <10 for both Glu and NAA were accepted.

### Manganese-Enhanced Magnetic Resonance Imaging (MEMRI)

MEMRI images were acquired on the same 7T Bruker ClinScan system with a Siemens console using a transmit-only whole-body coil and receive-only surface coil with established parameters (Bissig and Berkowitz, 2009, 2011; Perrine et al., 2015; Ouyang et al., 2017; Bosse et al., 2018). Twenty-four hours prior to each scan, rats received intraperitoneal (i.p.) injection of manganese (Mn^2+^; 66 mg/kg MnCl_2_·4H_2_O). Before each scan, animals were anesthetized with isoflurane (1%–5%, 0.4 L/min O_2_), and then placed in a prone position and secured as for ^1^H-MRS before being placed into the magnet’s horizontal bore. Rats were maintained on the same percent of isoflurane throughout the scan. Vital signs and cardiac gating were monitored, and body temperature was maintained at 37°C using a heated re-circulating water system located beneath the rat. Rapid acquisition gradient echo (MPRAGE) and proton density-weighted (PDGE) images were acquired sequentially using a dual coil mode per animal with principally mutual parameters (echo time = 3.03 ms, turbo factor = 9, echo spacing = 7.77 ms, field of view 2.50 × 2.50 × 2.91 cm^3^, matrix size 192 × 192 × 112, resulting a resolution of 130 μm × 130 μm × 260 μm, slice thickness 260 μm).

**Figure 2:**
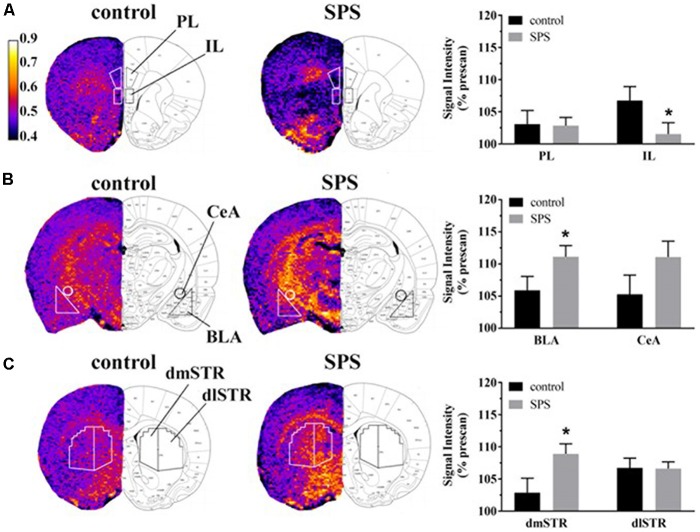
Effects of SPS on neural activity in multiple brain regions assessed with manganese (Mn^2+^)-enhanced MRI (MEMRI). Magnetization prepared rapid acquisition gradient echo/proton density weighted (MPRAGE/PDGE) images of coronal sections containing **(A)** the prelimbic (PL) and infralimbic (IL) cortices, **(B)** the basolateral (BLA) and central extended (CeA) amygdala, and **(C)** the dorsomedial (dmSTR) and dorsolateral (dlSTR) striatum. Each panel **(A–C)** from left to right shows: region of interest (ROI) placements on a representative MPRAGE/PDGE ratio image (pseudocolor indicates signal intensity, scale bar: lighter color indicates higher activity with arbitrary units and Mn^2+^ uptake) adjacent to the corresponding rat brain atlas image (adapted from Paxinos and Watson, 2007), and average normalized signal intensities plotted as % prescan (mean ± SEM) for each ROI (control *n* = 8, SPS *n* = 9). *p < 0.05 compared to controls.

T_1_-weighted images were generated by dividing the signal intensity of MPRAGE images with the corresponding PDGE images on a voxel-by-voxel basis (Bissig and Berkowitz, 2009, 2011; Perrine et al., 2015; Bosse et al., 2018). MPRAGE, PDGE, and T_1_-weighted ratio images were uploaded in ImageJ (Schneider et al., 2012) for ROI analysis. Construction of 2D ROI templates, representing PL/IL (Figure 2A, left), BLA/CeA (Figure 2B, left), and dmSTR/dlSTR (Figure 2C, left), were guided by neuroanatomical landmarks with careful comparison of MR images with a rat brain atlas (Paxinos and Watson, 2007). Prominent landmarks included white matter tracts (e.g., the genu of the corpus callosum, anterior and posterior commissures) as well as the overall brain and ventricle profile. The atlas-based, user-defined ROI templates were used to maintain uniform quantification of signal intensities from T_1_-weighted ratio images across subjects. Average signal intensities were measured using ImageJ for each ROI and normalized to the mean signal intensity recorded for the temporalis muscle tissue (located adjacent to the skull); mPFC and amygdala were also normalized within three consecutive brain slices. Prior to muscle normalization, signal intensities from lateral ROIs were averaged from the left and right hemisphere for each subject.

### Data and Statistical Analyses

The percent change from prescan (baseline) to postscan (post-SPS exposure) were calculated within each subject to acquire % prescan values for the ^1^H-MRS and MEMRI data, graphs and statistical comparisons. As the directionality of our data was predicted by previous studies (Knox et al., 2010; Perrine et al., 2016; Lim et al., 2017), ^1^H-MRS Glu and NAA data were analyzed by one-tailed Student’s *t*-tests and MEMRI signal intensities for each ROI were independently analyzed by one-tailed Student’s *t*-tests. Secondary analysis was also performed on the raw data using a 2 × 2 factorial design with repeated measures followed by multiple comparisons using Fischer’s LSD test. Data from both statistical designs are presented in the results text, and statistical results presented in the figures focus on the primary analysis. Statistical analyses were performed using GraphPad Prism 8 (GraphPad Software, Inc, La Jolla, CA, USA) and criterion for statistical significance was *p* < 0.05.

## Results

^1^H-MRS was used to quantify Glu levels in the mPFC and dSTR (Figure 1). Figures 1A,B (left) shows voxel placements in the mPFC and dSTR, respectively. Figure 1A (middle) shows a representative spectrum from the mPFC with insets illustrating the Glu spectra from (middle-left) a control rat and (middle-right) an SPS-exposed rat. Figure 1B (middle) shows a representative spectrum from the STR with insets illustrating the NAA spectra from a control rat (middle-left) and an SPS-exposed rat (middle-right). As hypothesized and previously shown (Knox et al., 2010; Perrine et al., 2016; Lim et al., 2017), SPS-exposed rats had significantly decreased Glu levels (as % prescan) in the mPFC compared to control rats [Figure 1A (right); control *n* = 5, SPS *n* = 4; (*t*_(7)_ = 3.59, *p* = 0.004)], and secondary analysis revealed a main effect interaction between stress exposure and scan-time *F*_(1,7)_ = 14.13 (*p* = 0.007) and *post hoc* effect showing SPS significantly decreased Glu in mPFC from prescan to postscan (*p* = 0.007) and a trend for a decrease in mPFC Glu postscan values in SPS-exposed rats compared to controls (*p* = 0.060). In the dSTR, SPS-exposed rats displayed an increase in NAA levels compared to controls [Figure 1B (right); control *n* = 4, SPS *n* = 5; (*t*_(7)_ = 2.655, *p* = 0.016)]. Secondary analysis did not reveal a main effect, but *post hoc* analysis showed a significant increase in striatal NAA between SPS and control postscan values (*p* = 0.023) and a trend for an increase in striatal NAA in SPS-exposed rats from prescan to postscan (*p* = 0.056). Tables s 1, 2 summarize the prescan (baseline, before-exposure) and postscan (after SPS or control exposure) values of these ^1^H-MRS metabolites of interest in the mPFC and dSTR, respectively.

**Table 1:** Summary of metabolites in the medial prefrontal cortex (mPFC) as measured by proton magnetic resonance spectroscopy (^1^H-MRS).

	Control		SPS	
Metabolite	Prescan	Postscan	Prescan	Postscan
Glutamate	7.56 ± 0.53	8.31 ± 0.29	8.92 ± 0.52	6.75 ± 0.84
N-acetylaspartate	6.15 ± 0.59	5.89 ± 0.11	6.03 ± 0.31	5.29 ± 0.32

*Prescan and postscan values reported as mean ± SEM (arbitrary units) for control (*n* = 5) and single-prolonged stress (SPS)-exposed rats (*n* = 4)*.

**Table 2:** Summary of metabolites in the dorsal striatum (dSTR) as measured by ^1^H-MRS.

	Control		SPS	
Metabolite	Prescan	Postscan	Prescan	Postscan
Glutamate	6.18 ± 0.47	6.64 ± 0.30	6.70 ± 0.53	6.54 ± 0.58
N-acetylaspartate	4.71 ± 0.07	4.49 ± 0.22	4.77 ± 0.31	5.60 ± 0.30

*Prescan and postscan values reported as mean ± SEM (arbitrary units) for control (*n* = 4) and SPS-exposed rats (*n* = 5)*.

Neural activity was assessed in SPS-exposed and control rats using MEMRI (Figure 2). Representative images depicting ROIs used to quantify Mn^2+^ uptake are shown in Figures 2A–C from control (left, *n* = 8) and SPS exposed rats (middle, *n* = 9) alongside corresponding data (right) reported as % prescan. Within the mPFC subregions, MEMRI results indicate that neural activity, normalized to prescan values, was unchanged in SPS-exposed rats in the PL (*t*_(15)_ = 0.09, *p* = 0.46), but decreased in the IL (*t*_(15)_ = 1.89, *p* = 0.039), compared to control rats (Figure 2A). Secondary analysis of the IL neural activity data showed a main effect of scan-time *F*_(1,15)_ = 8.161 (*p* = 0.012) with *post hoc* analysis showing a trend for a decrease in IL neural activity in SPS-exposed rats compared to controls for postscan values (*p* = 0.058). Conversely, in amygdala subregions, SPS-exposed rats showed increased neural activity as % prescan in the BLA (*t*_(15)_ = 1.89, *p* = 0.039), but not in the CeA (*t*_(15)_ = 1.51, *p* = 0.075), compared to control rats (Figure 2B). Secondary analysis of the BLA neural activity data showed a main effect of scan-time *F*_(1,15)_ = 37.45 (*p* < 0.0001) with *post hoc* analysis revealing SPS significantly increased BLA neural activity from prescan to postscan (*p* < 0.0001). SPS-exposed rats also displayed increased neural activity, expressed relative to prescan levels, in the dmSTR (*t*_(15)_ = 2.25, *p* = 0.020), but not the dlSTR (*t*_(15)_ = 0.05, *p* = 0.47), compared to control rats (Figure 2C). Secondary analysis of the dSTR neural activity data showed a main effect of scan-time *F*_(1,15)_ = 37.45 (*p* = 0.001) with *post hoc* analysis revealing SPS significantly increased dSTR neural activity from prescan to postscan (*p* = 0.0003).

## Discussion

In this study, our first goal was to assess longitudinal Glu levels and NAA *in vivo* in the mPFC and the dSTR after SPS using ^1^H-MRS. The second goal was to determine if SPS changed longitudinal neural activity *in vivo* in subregions of the mPFC, amygdala, and STR using MEMRI. The *a priori* hypothesis of the current study was that SPS-exposed rats would show decreased Glu levels in the mPFC with increased neural activity in the PL, decreased neural activity in the IL, and increased neural activity in the BLA and dmSTR. As expected, and replicating our previous findings, we observed that animals exposed to SPS had decreased Glu levels in the mPFC (Knox et al., 2010; Perrine et al., 2016). We extend this finding by showing decreased neural activity in the IL, but not in the PL, cortices. Collectively, we parsimoniously synthesize these findings to suggest that SPS decreases Glu-based activity in the IL subregion of the mPFC. We also found increased neural activity in the amygdala after SPS, as anticipated based on human PTSD neuroimaging studies, and specifically, that this activity was significantly increased in the BLA, but not in the CeA. Our most novel findings were observed in the STR, where we showed that SPS increased NAA levels in the dSTR and increased neural activity in the dmSTR, both of which suggest increased neural hyperactivity in animals exposed to traumatic stress.

The PFC plays an important role in executive control and exerts “top-down” influence on the amygdala. The PFC-amygdala network mediates associative fear learning (Marek et al., 2013; Arruda-Carvalho and Clem, 2015), which is critical to both overgeneralization (Kaczkurkin et al., 2017) and extinction deficits seen in humans with PTSD (Rabinak et al., 2017). Clinical studies using functional MRI and positron-emission tomography consistently show hypoactivity in the ventromedial PFC and hyperactivity in the dorsolateral PFC and amygdala complex during fear conditioning in humans with PTSD (Bremner et al., 2005; Etkin and Wager, 2007; Milad et al., 2009; Rougemont-Bücking et al., 2011; Pitman et al., 2012). In animal studies, SPS-exposed rodents show extinction retention deficits after fear conditioning, decreased c-fos immunoreactivity in the IL following fear extinction and increased BLA neural activity during fear extinction training (Knox et al., 2012a, 2016; Perrine et al., 2016). Our results demonstrate that SPS decreases activity in the IL, but not the PL, would indicate a lack of IL-mediated activation of the CeA, which would be important to inhibit fear response after SPS and suppress amygdala output through the BLA as presently observed. Hyperactivity in BLA, as shown herein, would alter fear extinction, without an effect on fear acquisition (Sierra-Mercado et al., 2011). A previous study from our lab has demonstrated that SPS reduces Glu and glutamine in the mPFC 7 days after exposure as measured by ^1^H-MRS at 11.7T *ex vivo* (Knox et al., 2012a). Similar results have been reported in rats by another group (Lim et al., 2017) and in mice by our group (Perrine et al., 2016). These preclinical findings parallel human reports, where PTSD is associated with decreased Glu (Yang et al., 2015) in the ACC, which may predict hyperarousal symptoms (Meyerhoff et al., 2014). These effects appear to be brain-region-specific, because in temporal, parietal, occipital, and insular cortices, an increase in Glu is generally observed in individuals with PTSD (Averill et al., 2017). We show no change in PFC GABA levels (data not shown), which is consistent with a meta-analysis of ^1^H-MRS studies in humans with PTSD (Rosso et al., 2014; Schür et al., 2016); however, ^1^H-MRS studies in humans with PTSD have shown increased GABA levels in the ACC, but decreased GABA levels in other cortical areas (Michels et al., 2014; Averill et al., 2017). Finally, we observe no change in PFC NAA levels after SPS; whereas, albeit inconsistently reported, a reduction in NAA/creatine ratio in ACC has been observed in individuals with PTSD (Mahmutyazicioğlu et al., 2005; Ham et al., 2007; Schuff et al., 2008). This dysregulation in mPFC-amygdala neurocircuitry, leading to impaired prefrontal control of fear and emotion, is thought to underlie pathological fear responses in PTSD (Liberzon and Sripada, 2008). As indicated, our results match with previous preclinical and clinical PTSD studies that show decreased Glu levels in the mPFC. Our data further suggest that SPS affects Glu-based mPFC and amygdala subregions, which is reflected as IL hypoactivity and proposed to cause BLA hyperactivity.

Increasing evidence suggests that PTSD results in reward circuitry dysfunction. In those with PTSD, functional MRI studies show decreased striatal activation during a monetary reward task, indicating anhedonic behavior (Elman et al., 2009). In parallel rodent studies, Perrine and colleagues have shown that rats exposed to SPS demonstrate anhedonic behavior during a sucrose preference task and in cocaine self-administration studies, which was associated with decreased dopamine and dopamine 2 receptor levels in the STR (Enman et al., 2015). Similarly, studies from our group indicate that SPS-exposed mice show impaired behavioral sensitization to ethanol that is accompanied by decreased striatal dopamine 2 receptor levels and increased striatal postsynaptic density protein 95, a marker of increase neuronal plasticity (Matchynski-Franks et al., 2016). PTSD has also been associated with deficits in inhibitory control, suggesting that the dSTR is activated to support high-demand inhibitory processing (Falconer et al., 2008). These data support the hypothesis that changes in dSTR activation may be related to impaired inhibitory control observed following SPS. Similar to the amygdala, PFC also exerts “top-down” control of STR function. The dmSTR receives projections from the PL and is responsible for action-outcome association, whereas the dlSTR receives projections primarily from the IL and is responsible for habit-like behaviors (Moussa et al., 2011; Burton et al., 2015; Kalivas and Kalivas, 2016; Kaczkurkin et al., 2017; Ma et al., 2017). Our results show that SPS decreases activity in the IL and increases activity in the dmSTR, but not dlSTR, which may suggest that SPS affects action-outcome association, but not habit-like behaviors. In the current study, dSTR Glu levels did not change, however NAA levels increased in the dSTR after SPS exposure. No studies have yet measured NAA levels in dSTR in humans with PTSD, adding novelty to the present findings. A clinical study demonstrating a reduction in NAA within the ACC and hippocampus of patients with PTSD interpreted this change as indicating a disruption of neuronal integrity (Ham et al., 2007), suggesting in our study that SPS influenced dSTR neuronal integrity without affecting its excitatory tone.

In conclusion, results from the present study corroborate results from clinical and pre-clinical studies. We show that SPS-exposed rats have decreased glutamate in the mPFC with decreased neural activity in the IL and increased neural activity in the BLA. Our novel findings in this study show that traumatic stress also affects reward neurocircuitry in the dSTR with increased neural activity and neuronal integrity in the dmSTR, confirming that coordinated function across the neurocircuitry of mPFC, STR, and amygdala is important in the pathology of PTSD. Our data indicating that the presently used animal model of traumatic stress recapitulates several neurochemical features of clinical PTSD. These findings support its utility to explore therapeutic interventions aimed at mitigating the aberrant functionality of these regions and to gain further mechanistic insight into brain-behavior abnormalities of PTSD.

## Author Contributions

SP designed the experiments. SP, FG, and ML performed the experiments with technical input from JSta. VP, ML, JStr and KB analyzed the data. SP and AC reviewed the data and interpreted findings. VP and KB drafted the manuscript. All other authors provided critical input and revisions.

## Conflict of Interest Statement

The authors declare that the research was conducted in the absence of any commercial or financial relationships that could be construed as a potential conflict of interest.

## Abbreviations

^1^H-MRS: proton magnetic resonance spectroscopy
ACC: anterior cingulate cortex
BLA: basolateral amygdala
CeA: central nucleus of the amygdala
dl STR: dorsolateral striatum
dmSTR: dorsomedial striatum
dSTR: dorsal striatum
IL: infralimbic
MEMRI: manganese-enhanced magnetic resonance imaging
mPFC: medial prefrontal cortex
MPRAGE: rapid acquisition gradient echo
NAA: N-acetylaspartate
PDGE: proton density-weighted
PFC: prefrontal cortex
PL: prelimbic
PTSD: post-traumatic stress disorder
ROI: region of interest
SPS: single prolonged stress
STR: striatum.

## References

[B1] Arruda-Carvalho M. Clem R. L. (2015). Prefrontal-amygdala fear networks come into focus. Front. Syst. Neurosci. 9:145. 10.3389/fnsys.2015.00145 26578902 PMC4626554

[B2] Averill L. A. Purohit P. Averill C. L. Boesl M. A. Krystal J. H. Abdallah C. G. (2017). Glutamate dysregulation and glutamatergic therapeutics for PTSD: evidence from human studies. Neurosci. Lett. 649, 147–155. 10.1016/j.neulet.2016.11.064 27916636 PMC5482215

[B3] Bissig D. Berkowitz B. A. (2009). Manganese-enhanced MRI of layer-specific activity in the visual cortex from awake and free-moving rats. Neuroimage 44, 627–635. 10.1016/j.neuroimage.2008.10.013 19015035 PMC2642584

[B4] Bissig D. Berkowitz B. A. (2011). Same-session functional assessment of rat retina and brain with manganese-enhanced MRI. Neuroimage 58, 749–760. 10.1016/j.neuroimage.2011.06.062 21749922 PMC3787253

[B5] Blanco C. Xu Y. Brady K. Pérez-Fuentes G. Okuda M. Wang S. (2013). Comorbidity of posttraumatic stress disorder with alcohol dependence among US adults: results from national epidemiological survey on alcohol and related conditions. Drug Alcohol. Depend. 132, 630–638. 10.1016/j.drugalcdep.2013.04.016 23702490 PMC3770804

[B6] Bosse K. E. Ghoddoussi F. Eapen A. T. Charlton J. L. Susick L. L. Desai K.. (2018). Calcium/calmodulin-stimulated adenylyl cyclases 1 and 8 regulate reward-related brain activity and ethanol consumption. Brain Imaging Behav. [Epub ahead of print]. 10.1007/s11682-018-9856-6 29594872 PMC6202255

[B7] Brand L. Groenewald I. Stein D. J. Wegener G. Harvey B. H. (2008). Stress and re-stress increases conditioned taste aversion learning in rats: possible frontal cortical and hippocampal muscarinic receptor involvement. Eur. J. Pharmacol. 586, 205–211. 10.1016/j.ejphar.2008.03.004 18439577

[B8] Bremner J. D. Vermetten E. Schmahl C. Vaccarino V. Vythilingam M. Afzal N.. (2005). Positron emission tomographic imaging of neural correlates of a fear acquisition and extinction paradigm in women with childhood sexual-abuse-related post-traumatic stress disorder. Psychol. Med. 35, 791–806. 10.1017/s0033291704003290 15997600 PMC3233760

[B9] Burton A. C. Nakamura K. Roesch M. R. (2015). From ventral-medial to dorsal-lateral striatum: neural correlates of reward-guided decision-making. Neurobiol. Learn. Mem. 117, 51–59. 10.1016/j.nlm.2014.05.003 24858182 PMC4240773

[B10] Do-Monte F. H. Manzano-Nieves G. Quiñones-Laracuente K. Ramos-Medina L. Quirk G. J. (2015). Revisiting the role of infralimbic cortex in fear extinction with optogenetics. J. Neurosci. 35, 3607–3615. 10.1523/JNEUROSCI.3137-14.2015 25716859 PMC4339362

[B11] Eagle A. L. Fitzpatrick C. J. Perrine S. A. (2013). Single prolonged stress impairs social and object novelty recognition in rats. Behav. Brain Res. 256, 591–597. 10.1016/j.bbr.2013.09.014 24036168 PMC3857706

[B12] Eagle A. L. Singh R. Kohler R. J. Friedman A. L. Liebowitz C. P. Galloway M. P.. (2015). Single prolonged stress effects on sensitization to cocaine and cocaine self-administration in rats. Behav. Brain Res. 284, 218–224. 10.1016/j.bbr.2015.02.027 25712697 PMC5370568

[B13] Elman I. Lowen S. Frederick B. B. Chi W. Becerra L. Pitman R. K. (2009). Functional neuroimaging of reward circuitry responsivity to monetary gains and losses in posttraumatic stress disorder. Biol. Psychiatry 66, 1083–1090. 10.1016/j.biopsych.2009.06.006 19640506 PMC9446383

[B14] Enman N. M. Arthur K. Ward S. J. Perrine S. A. Unterwald E. M. (2015). Anhedonia, reduced cocaine reward and dopamine dysfunction in a rat model of posttraumatic stress disorder. Biol. Psychiatry 78, 871–879. 10.1016/j.biopsych.2015.04.024 26115790 PMC4644715

[B15] Etkin A. Wager T. D. (2007). Functional neuroimaging of anxiety: a meta-analysis of emotional processing in PTSD, social anxiety disorder and specific phobia. Am. J. Psychiatry 164, 1476–1488. 10.1176/appi.ajp.2007.07030504 17898336 PMC3318959

[B16] Everitt B. J. Robbins T. W. (2016). Drug addiction: updating actions to habits to compulsions ten years on. Annu. Rev. Psychol. 67, 23–50. 10.1146/annurev-psych-122414-033457 26253543

[B17] Falconer E. Bryant R. Felmingham K. L. Kemp A. H. Gordon E. Peduto A.. (2008). The neural networks of inhibitory control in posttraumatic stress disorder. J. Psychiatry Neurosci. 33, 413–422. 18787658 PMC2527717

[B18] Ganon-Elazar E. Akirav I. (2012). Cannabinoids prevent the development of behavioral and endocrine alterations in a rat model of intense stress. Neuropsychopharmacology 37, 456–466. 10.1038/npp.2011.204 21918506 PMC3242307

[B19] Gruetter R. Tkác I. (2000). Field mapping without reference scan using asymmetric echo-planar techniques. Magn. Reson. Med. 43, 319–323. 10.1002/(sici)1522-2594(200002)43:2<319::aid-mrm22>3.3.co;2-t 10680699

[B20] Ham B. J. Chey J. Yoon S. J. Sung Y. Jeong D. U. Ju Kim S.. (2007). Decreased N-acetyl-aspartate levels in anterior cingulate and hippocampus in subjects with post-traumatic stress disorder: a proton magnetic resonance spectroscopy study. Eur. J. Neurosci. 25, 324–329. 10.1111/j.1460-9568.2006.05253.x 17241294

[B21] Hayes J. P. Hayes S. M. Mikedis A. M. (2012). Quantitative meta-analysis of neural activity in posttraumatic stress disorder. Biol. Mood Anxiety Disord. 2:9. 10.1186/2045-5380-2-9 22738125 PMC3430553

[B22] Kaczkurkin A. N. Burton P. C. Chazin S. M. Manbeck A. B. Espensen-Sturges T. Cooper S. E.. (2017). Neural substrates of overgeneralized conditioned fear in PTSD. Am. J. Psychiatry 174, 125–134. 10.1176/appi.ajp.2016.15121549 27794690 PMC7269602

[B23] Kalivas B. C. Kalivas P. W. (2016). Corticostriatal circuitry in regulating diseases characterized by intrusive thinking. Dialogues Clin. Neurosci. 18, 65–76. 27069381 10.31887/DCNS.2016.18.1/pkalivas PMC4826772

[B24] Khan S. Liberzon I. (2004). Topiramate attenuates exaggerated acoustic startle in an animal model of PTSD. Psychopharmacology 172, 225–229. 10.1007/s00213-003-1634-4 14586539

[B25] Knox D. George S. A. Fitzpatrick C. J. Rabinak C. A. Maren S. Liberzon I. (2012a). Single prolonged stress disrupts retention of extinguished fear in rats. Learn. Mem. 19, 43–49. 10.1101/lm.024356.111 22240323 PMC3262971

[B26] Knox D. Nault T. Henderson C. Liberzon I. (2012b). Glucocorticoid receptors and extinction retention deficits in the single prolonged stress model. Neuroscience 223, 163–173. 10.1016/j.neuroscience.2012.07.047 22863672

[B27] Knox D. Perrine S. A. George S. A. Galloway M. P. Liberzon I. (2010). Single prolonged stress decreases glutamate, glutamine and creatine concentrations in the rat medial prefrontal cortex. Neurosci. Lett. 480, 16–20. 10.1016/j.neulet.2010.05.052 20546834 PMC2902659

[B28] Knox D. Stanfield B. R. Staib J. M. David N. P. Keller S. M. DePietro T. (2016). Neural circuits via which single prolonged stress exposure leads to fear extinction retention deficits. Learn. Mem. 23, 689–698. 10.1101/lm.043141.116 27918273 PMC5110987

[B29] Li X. Han F. Liu D. Shi Y. (2010). Changes of Bax, Bcl-2 and apoptosis in hippocampus in the rat model of post-traumatic stress disorder. Neurol. Res. 32, 579–586. 10.1179/016164110X12556180206194 20092675

[B31] Liberzon I. Krstov M. Young E. A. (1997). Stress-restress: effects on ACTH and fast feedback. Psychoneuroendocrinology 22, 443–453. 10.1016/s0306-4530(97)00044-9 9364622

[B32] Liberzon I. López J. F. Flagel S. B. Vázquez D. M. Young E. A. (1999). Differential regulation of hippocampal glucocorticoid receptors mRNA and fast feedback: relevance to post-traumatic stress disorder. J. Neuroendocrinol. 11, 11–17. 10.1046/j.1365-2826.1999.00288.x 9918224

[B30] Liberzon I. Sripada C. S. (2008). The functional neuroanatomy of PTSD: a critical review. Prog. Brain Res. 167, 151–169. 10.1016/s0079-6123(07)67011-3 18037013

[B33] Lim S. I. Song K. H. Yoo C. H. Woo D. C. Choe B. Y. (2017). Decreased glutamatergic activity in the frontal cortex of single prolonged stress model: *in vivo* and *ex vivo* proton MR spectroscopy. Neurochem. Res. 42, 2218–2229. 10.1007/s11064-017-2232-x 28349360

[B34] Lisieski M. J. Eagle A. L. Conti A. C. Liberzon I. Perrine S. A. (2018). Single-prolonged stress: a review of two decades of progress in a rodent model of post-traumatic stress disorder. Front. Psychiatry 9:196. 10.3389/fpsyt.2018.00196 29867615 PMC5962709

[B35] Ma T. Barbee B. Wang X. Wang J. (2017). Alcohol induces input-specific aberrant synaptic plasticity in the rat dorsomedial striatum. Neuropharmacology 123, 46–54. 10.1016/j.neuropharm.2017.05.014 28526611 PMC5531610

[B36] Mahmutyazicioğlu K. Konuk N. Ozdemir H. Atasoy N. Atik L. Gündoğdu S. (2005). Evaluation of the hippocampus and the anterior cingulate gyrus by proton MR spectroscopy in patients with post-traumatic stress disorder. Diagn. Interv. Radiol. 11, 125–129. 16206051

[B37] Marek R. Strobel C. Bredy T. W. Sah P. (2013). The amygdala and medial prefrontal cortex: partners in the fear circuit. J. Physiol. 591, 2381–2391. 10.1113/jphysiol.2012.248575 23420655 PMC3678031

[B38] Matchynski-Franks J. J. Susick L. L. Schneider B. L. Perrine S. A. Conti A. C. (2016). Impaired ethanol-induced sensitization and decreased cannabinoid receptor-1 in a model of posttraumatic stress disorder. PLoS One 11:e0155759. 10.1371/journal.pone.0155759 27186643 PMC4871361

[B39] Meyerhoff D. J. Mon A. Metzler T. Neylan T. C. (2014). Cortical gamma-aminobutyric acid and glutamate in posttraumatic stress disorder and their relationships to self-reported sleep quality. Sleep 37, 893–900. 10.5665/sleep.3654 24790267 PMC3985106

[B40] Michels L. Schulte-Vels T. Schick M. O’Gorman R. L. Zeffiro T. Hasler G.. (2014). Prefrontal GABA and glutathione imbalance in posttraumatic stress disorder: preliminary findings. Psychiatry Res. 224, 288–295. 10.1016/j.pscychresns.2014.09.007 25448399

[B41] Milad M. R. Pitman R. K. Ellis C. B. Gold A. L. Shin L. M. Lasko N. B.. (2009). Neurobiological basis of failure to recall extinction memory in posttraumatic stress disorder. Biol. Psychiatry 66, 1075–1082. 10.1037/e717692011-019 19748076 PMC2787650

[B42] Milad M. R. Rauch S. L. Pitman R. K. Quirk G. J. (2006). Fear extinction in rats: implications for human brain imaging and anxiety disorders. Biol. Psychol. 73, 61–71. 10.1016/j.biopsycho.2006.01.008 16476517

[B43] Moussa R. Poucet B. Amalric M. Sargolini F. (2011). Contributions of dorsal striatal subregions to spatial alternation behavior. Learn. Mem. 18, 444–451. 10.1101/lm.2123811 21685151

[B44] Ouyang J. Pace E. Lepczyk L. Kaufman M. Zhang J. Perrine S. A.. (2017). Blast-induced tinnitus and elevated central auditory and limbic activity in rats: a manganese-enhanced mri and behavioral study. Sci. Rep. 7:4852. 10.1038/s41598-017-04941-w 28687812 PMC5501813

[B45] Paxinos G. Watson C. (2007). The Rat Brain in Stereotaxic Coordinates. Boston: Elsevier. 10.1016/0165-0270(80)90021-7 6110810

[B46] Perrine S. A. Eagle A. L. George S. A. Mulo K. Kohler R. J. Gerard J.. (2016). Severe, multimodal stress exposure induces PTSD-like characteristics in a mouse model of single prolonged stress. Behav. Brain Res. 303, 228–237. 10.1016/j.bbr.2016.01.056 26821287

[B47] Perrine S. A. Ghoddoussi F. Desai K. Kohler R. J. Eapen A. T. Lisieski M. J.. (2015). Cocaine-induced locomotor sensitization in rats correlates with nucleus accumbens activity on manganese-enhanced MRI. NMR Biomed. 28, 1480–1488. 10.1002/nbm.3409 26411897 PMC4618766

[B48] Peters J. Kalivas P. W. Quirk G. J. (2009). Extinction circuits for fear and addiction overlap in prefrontal cortex. Learn. Mem. 16, 279–288. 10.1101/lm.1041309 19380710 PMC4527308

[B49] Pitman R. K. Rasmusson A. M. Koenen K. C. Shin L. M. Orr S. P. Gilbertson M. W.. (2012). Biological studies of post-traumatic stress disorder. Nat. Rev. Neurosci. 13, 769–787. 10.1038/nrn3339 23047775 PMC4951157

[B50] Rabinak C. A. Mori S. Lyons M. Milad M. R. Phan K. L. (2017). Acquisition of CS-US contingencies during Pavlovian fear conditioning and extinction in social anxiety disorder and posttraumatic stress disorder. J. Affect. Disord. 207, 76–85. 10.1016/j.jad.2016.09.018 27716541 PMC6642659

[B51] Rosso I. M. Weiner M. R. Crowley D. J. Silveri M. M. Rauch S. L. Jensen J. E. (2014). Insula and anterior cingulate GABA levels in posttraumatic stress disorder: preliminary findings using magnetic resonance spectroscopy. Depress. Anxiety 31, 115–123. 10.1002/da.22155 23861191 PMC3894264

[B52] Rougemont-Bücking A. Linnman C. Zeffiro T. A. Zeidan M. A. Lebron-Milad K. Rodriguez-Romaguera J.. (2011). Altered processing of contextual information during fear extinction in PTSD: an fMRI study. CNS Neurosci. Ther. 17, 227–236. 10.1111/j.1755-5949.2010.00152.x 20406268 PMC6493793

[B53] Sailer U. Robinson S. Fischmeister F. P. König D. Oppenauer C. Lueger-Schuster B.. (2008). Altered reward processing in the nucleus accumbens and mesial prefrontal cortex of patients with posttraumatic stress disorder. Neuropsychologia 46, 2836–2844. 10.1016/j.neuropsychologia.2008.05.022 18597797

[B54] Schneider C. A. Rasband W. S. Eliceiri K. W. (2012). NIH image to ImageJ: 25 years of image analysis. Nat. Methods 9, 671–675. 10.1038/nmeth.2089 22930834 PMC5554542

[B55] Schuff N. Neylan T. C. Fox-Bosetti S. Lenoci M. Samuelson K. Studholme C.. (2008). Abnormal N-acetylaspartate in hippocampus and anterior cingulate in posttraumatic stress disorder. Psychiatry Res. 162, 147–157. 10.1016/j.pscychresns.2007.04.011 18201876 PMC2443727

[B56] Schür R. R. Draisma L. W. Wijnen J. P. Boks M. P. Koevoets M. G. Joels M.. (2016). Brain GABA levels across psychiatric disorders: a systematic literature review and meta-analysis of ^1^H-MRS studies. Hum. Brain Mapp. 37, 3337–3352. 10.1002/hbm.23244 27145016 PMC6867515

[B57] Shin L. M. Liberzon I. (2010). The neurocircuitry of fear, stress and anxiety disorders. Neuropsychopharmacology 35, 169–191. 10.1038/npp.2009.83 19625997 PMC3055419

[B58] Sierra-Mercado D. Padilla-Coreano N. Quirk G. J. (2011). Dissociable roles of prelimbic and infralimbic cortices, ventral hippocampus and basolateral amygdala in the expression and extinction of conditioned fear. Neuropsychopharmacology 36, 529–538. 10.1038/npp.2010.184 20962768 PMC3005957

[B60] Wang W. Liu Y. Zheng H. Wang H. N. Jin X. Chen Y. C.. (2008). A modified single-prolonged stress model for post-traumatic stress disorder. Neurosci. Lett. 441, 237–241. 10.1016/j.neulet.2008.06.031 18577419

[B59] Wang H.-N. Peng Y. Tan Q.-R. Chen Y.-C. Zhang R.-G. Qiao Y.-T.. (2010). Quetiapine ameliorates anxiety-like behavior and cognitive impairments in stressed rats: implications for the treatment of posttraumatic stress disorder. Physiol. Res. 59, 263–271. 19537923 10.33549/physiolres.931756

[B61] Yang Z. Y. Quan H. Peng Z. L. Zhong Y. Tan Z. J. Gong Q. Y. (2015). Proton magnetic resonance spectroscopy revealed differences in the glutamate + glutamine/creatine ratio of the anterior cingulate cortex between healthy and pediatric post-traumatic stress disorder patients diagnosed after 2008 Wenchuan earthquake. Psychiatry Clin. Neurosci. 69, 782–790. 10.1111/pcn.12332 26171979

